# Deferred Lateral Margin Control in the Surgical Treatment of Genital Paget’s Disease and Lentiginous Vulvar Melanoma

**DOI:** 10.3390/jcm14010069

**Published:** 2024-12-26

**Authors:** Pedro Redondo

**Affiliations:** Department of Dermatology, University Clinic of Navarra, 28027 Madrid, Spain; predondo@unav.es; Tel.: +34-(94)-8255400

**Keywords:** deferred Mohs surgery, Spaguetti technique, extramammmary Paget’s disease, lentiginous vulvar melanoma, paraffin

## Abstract

**Background/Objectives:** Some skin tumors can extend beyond their clinical appearance. This presents an additional challenge, especially when the affected area is the genital region, which is more difficult for both the patient and the physician to access and monitor due to its location and anatomical characteristics. The treatment of these lesions is complex, and literature postulates Mohs surgery as the best therapeutic option. **Methods:** We describe our experience in two patients with the resection of vulvar lentiginous melanoma and genital extramammary Paget’s disease, using a method of deferred lateral margin control in the surgical treatment. **Results:** The “spaghetti technique”(ST) initially removing a small strip from all lateral margins of the lesion, which is then closed directly while awaiting the paraffin histological result. In a second stage, the tumors within those margins are removed, and immediate reconstruction is performed. The final oncological and functional result was satisfactory, with no notable side effects. **Conclusions:** This method is suited for large, poorly defined superficial tumors in the genital, perineal, and perianal regions, where a frozen section study would be slow and burdensome for the patient and surgeon. The ST preserves healthy tissue and can be performed by any surgeon and pathologist without additional training, and is more comfortable for patients, avoiding prolonged open wounds during multiple steps of tumor excision.

## 1. Introduction

Some skin tumors can extend beyond their clinical appearance. This presents an additional challenge, especially when the affected area is the genital region, which is more difficult for both the patient and the physician to access and monitor due to its location and anatomical characteristics. Approximately 2% of all melanomas in women occur in the vulva. Unilateral labial involvement is the most frequent anatomic distribution of disease, although extension across the midline and/or clitoral involvement occurs in approximately 30% of patients [1]. In a series of 100 patients, the histological type in 64 was acral lentiginous [2]. Vulvar melanoma is characterized by a high rate of local recurrence and metastasis, and surgical intervention remains the standard treatment for localized lesions, but the ideal surgical approach and optimal margins are still unclear. While radical vulvectomy increases morbidity, it does not improve survival rates or reduce recurrence when compared to wide local excision [3]. Factors such as Breslow depth, ulceration status, and achieving clear margins are significant predictors of vulvar melanoma-specific survival [4]. The Mohs micrographic surgery (MMS) technique is appealing as it allows for stepwise confirmation of clear margins, potentially eliminating the need for radical excision or vulvectomy [5]. The lentiginous component of vulvar melanoma can be very subtle, with minimal pigmentation and poorly defined peripheral margins, often leading to an underestimation of its extent and an increased risk of incomplete resection. The same issue occurs with lentigo maligna on the head and neck, as well as with the lentiginous component of acral melanoma. Classically, for the treatment of lentigo maligna melanoma (LMM) and the lentiginous component of acral melanoma, a margin of at least 10 mm is recommended to ensure complete excision [6]. The vulvar region has aesthetic and functional considerations that may lead to a tendency to minimize resection—either consciously or unconsciously—thereby increasing the risk of insufficient margins. Consequently, recurrences are frequent unless precise lateral margin control is employed, often using MMS techniques [7,8]. Differentiating between a tumoral or dysplastic melanocyte and a normal melanocyte usually requires additional immunohistochemical studies beyond hematoxylin–eosin staining. Recent studies have validated rapid immunohistochemical techniques for melanoma and lentigo maligna in MMS with frozen sections, although these techniques require specific training and are generally not considered cost-effective [9]. Mohs surgery using paraffin sections takes days or weeks, during which time open, wounds in the genital region are at risk of infection and can have significant functional and psychological impacts.

Extramammary Paget’s disease (EMPD) is an intraepithelial neoplasia that most frequently occurs in the anogenital region of older men and women. It is an apocrine adenocarcinoma that can spread epidermotropically for many years without invasive growth or metastasis. Due to various clinical presentations, diagnosis and treatment are often delayed. For a long time, wide local excision has been the preferred surgical treatment for EMPD, although it has recurrence rates of up to 60%, likely due to poor clinical margin definition and its multifocal nature with diffuse subclinical spread. Additionally, wide excision margins can result in large defects that cause anatomical deformity and morbidity. Mohs surgery is the treatment of choice, with a lower recurrence rate than conventional margin surgery [10,11,12,13]. However, since these lesions are often large, MMS can be time-consuming and logistically challenging, requiring well-trained surgeons, and is still associated with high recurrence rates. Non-surgical more conservative treatments for EMPD include topical imiquimod, 5-fluorouracil, radiotherapy, and photodynamic therapy [14].

The “spaghetti technique” (ST) and its variants involve initially removing a small strip from all lateral margins of the lesion, which is then closed directly while awaiting the paraffin histological result [15]. The process can be extended with additional lateral excisions until all margins are clear. In the second stage, the tumor within those margins is removed, and immediate reconstruction is performed. We describe our experience with the resection of vulvar lentiginous melanoma and genital extramammary Paget’s disease using a method of deferred lateral margin control in surgical treatment.

## 2. Materials and Methods

### 2.1. Case Reports

**Figure 1:**
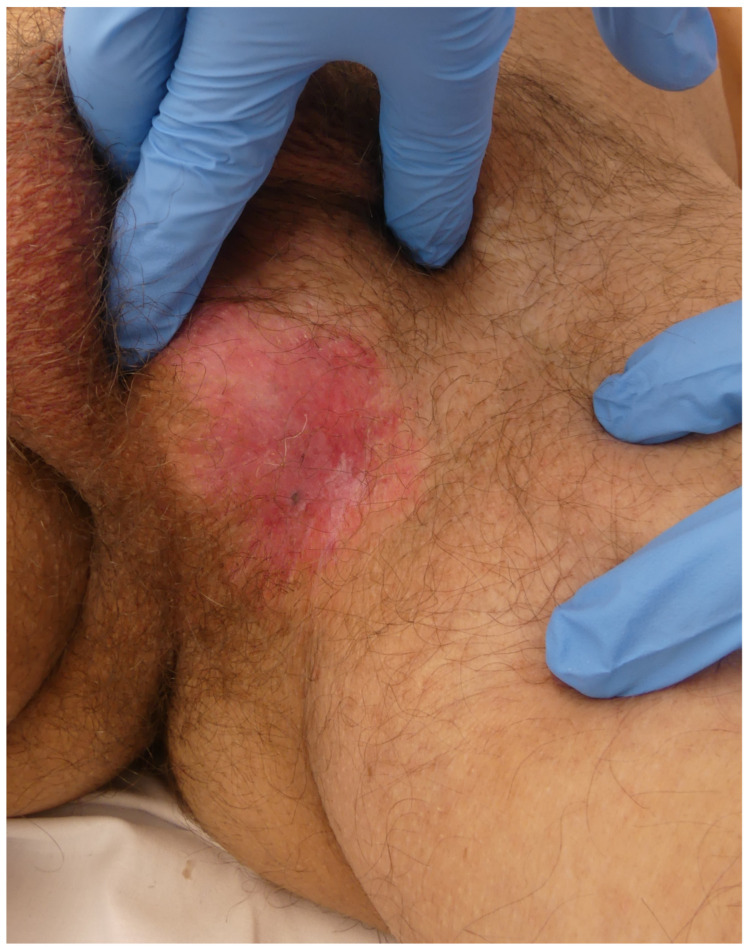
Ill-defined erythematous inguino-scrotal plaque with whitish areas.

**Figure 2:**
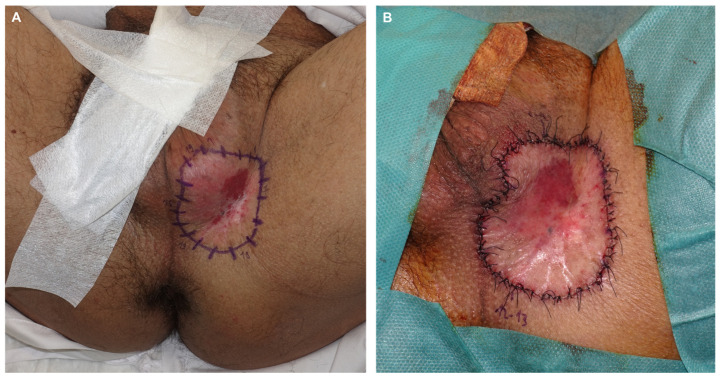
Design for excision with a 1 cm margin of healthy skin beyond the clinical edge (**A**). Immediate result following the first surgery. Nineteen lateral margins were evaluated. Closed with 5/0 silk, leaving longer sutures to delineate the transition between the edges (**B**).

**Figure 3:**
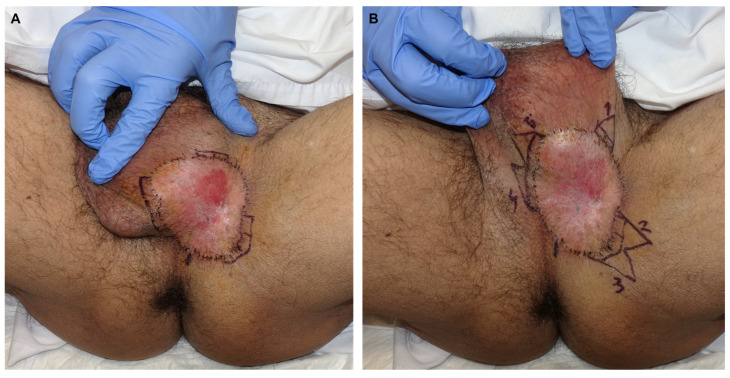
Minimal tumor involvement at margins 3, 9, 10, 15, and 16 after histological examination (**A**) Five Burrow triangles (1–5) are drawn on the affected margins for final closure of the defect (**B**).

**Figure 4:**
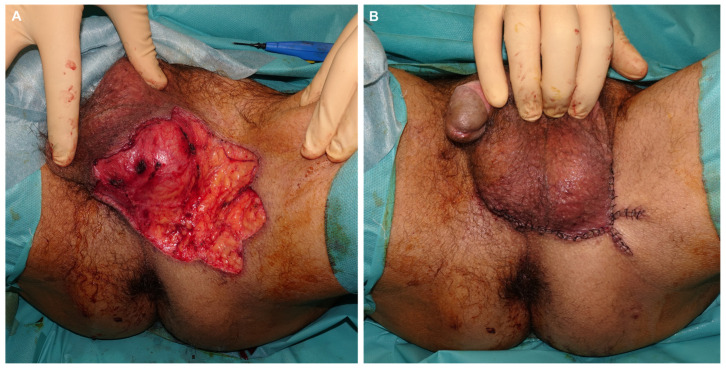
Final defect after excision of the central specimen and Burrow triangles. The latter is sent to the pathology lab oriented for subsequent paraffin study (**A**). Immediate result after direct closure utilizing scrotal skin, with 3 and 4/0 of 910 polyglactin braided suture and 5/0 silk (**B**).

**Figure 5:**
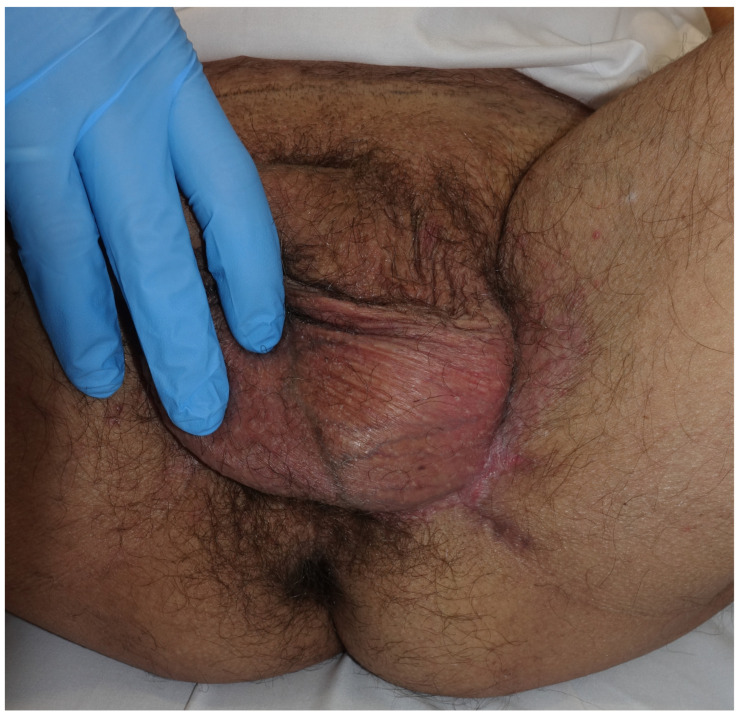
Appearance 4 months post-surgery.

Patient 1: A 59-year-old man presented with a pruritic lesion in the inguinoscrotal region, present for several months. Previous treatments with topical corticosteroids and antifungals had been ineffective. Physical examination revealed an erythematous plaque with whitish oval-shaped areas and diffuse borders (Figure 1). A punch biopsy confirmed EMPD. Treatment involved paraffin MMS using the ST. After defining the clinical margins, 1 cm of healthy skin was added, and a total of 19 oriented lateral margins were excised, closing the defects with 5/0 silk (Figure 2). Six days later, with five margins showing focal involvement, a new excision with five Burow’s triangles was performed for lateral margin assessment in paraffin, including the entire tumor (Figure 3). The defect was sutured in layers, with scrotal skin displaced using the release triangles (Figure 4). During a 2-year follow-up, no signs of local recurrence were observed (Figure 5).

**Figure 6:**
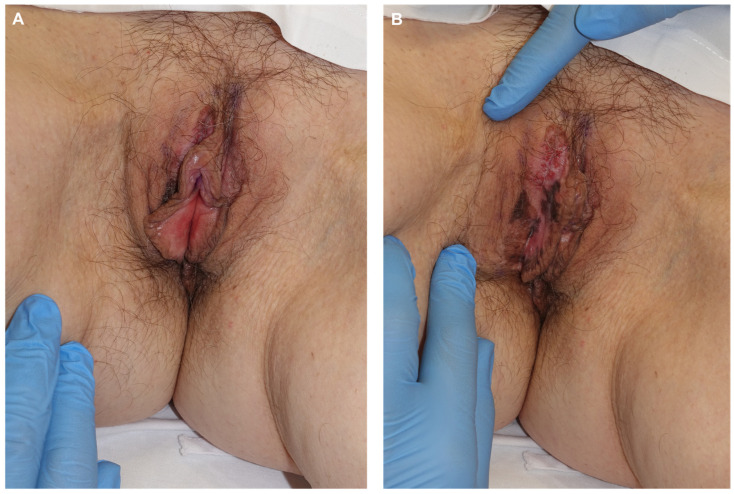
(**A,B**) Pigmented patchy lesion with whitish areas and minimally eroded sections, located on the external cutaneous border of the right labium majus.

**Figure 7:**
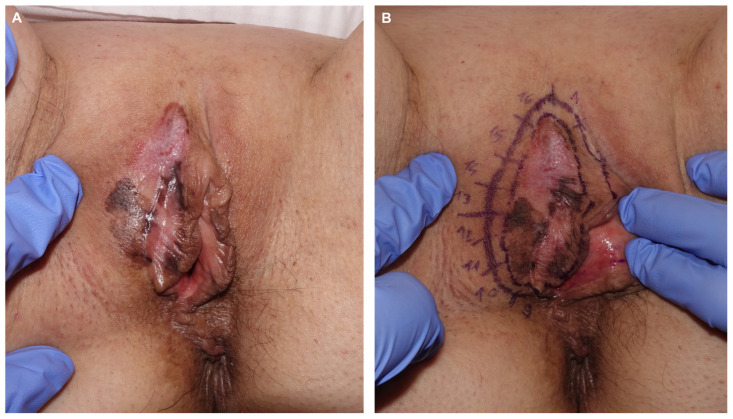
Clinically better-defined lesion after shaving the area (**A**). Delimitation of the initial excision of 16 lateral margins with at least 5 mm of healthy skin, confirmed via Wood’s light examination (**B**).

**Figure 8:**
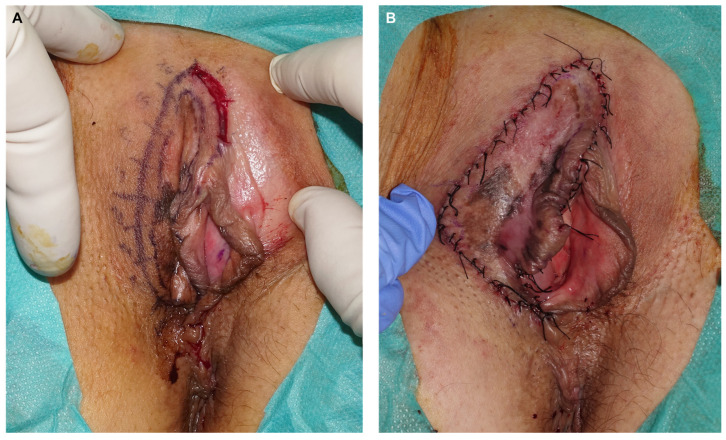
Example of the excision of margins 1 and 2 (**A**). Final outcome after the first surgery. Longer tails were left to delineate the margins from one another (**B**).

**Figure 9:**
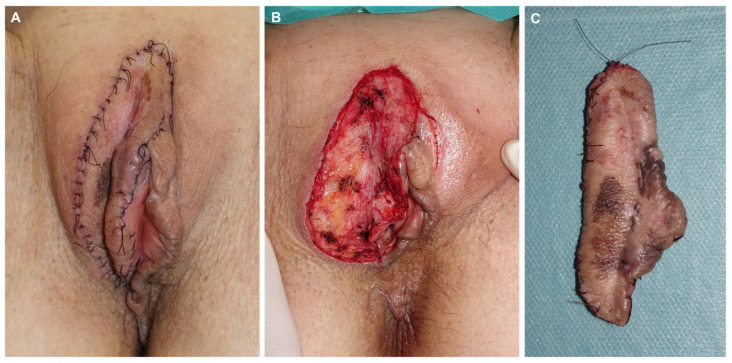
Appearance one week later, with all margins free after evaluation with hematoxylin–eosin and immunohistochemistry (**A**). Final defect after complete excision (**B**). Appearance of the final specimen sent to the pathology lab for definitive assessment (**C**).

**Figure 10:**
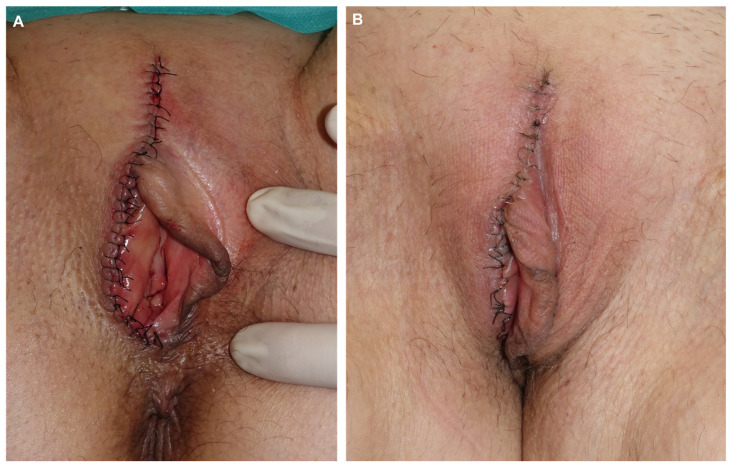
Final result after direct closure using 4/0 of 910 polyglactin braided suture and 5/0 silk (**A**). Appearance one week later before suture removal (**B**).

**Figure 11:**
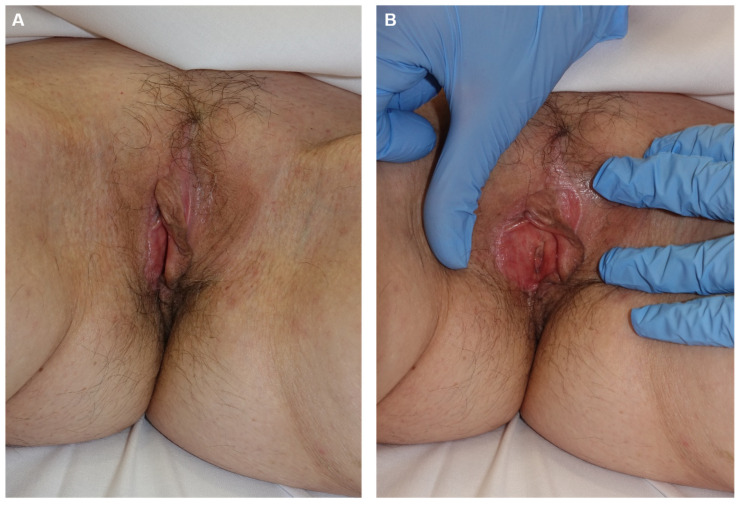
Appearance 4 months post-surgery (**A**,**B**).

Patient 2: A 64-year-old woman sought evaluation for pigmentation on the vulva with unclear onset and mild local pruritus. Examination revealed a diffuse plaque on the right labium majus with pigmented, achromic, and erythematous areas (Figure 6). Biopsies from three suspicious areas confirmed melanoma in situ. Treatment involved paraffin MMS using the ST (Figure 7). In the first procedure, lateral margins were excised after defining the lesion with Wood’s light and adding a 5 mm healthy skin margin (Figure 8). A week later, with clear margins confirmed by conventional study and immunohistochemistry with Melan A and PRAME, a second procedure was performed to excise the bulk of the lesion and close the defect directly (Figure 9 and Figure 10). The final diagnosis of the entire excised specimen was melanoma in situ, with a small area of vertical growth measuring 0.36 mm Breslow. During a 6-month follow-up, no signs of local recurrence were observed (Figure 11).

### 2.2. Surgical Technique

The technique involves studying the lateral margins in paraffin before excising the central piece containing the entire tumor. A double-blade scalpel can be used to remove a 1.5 to 2 mm strip around the lesion, which is then sutured. The sample is embedded in paraffin, and vertical sections containing 100% of the lateral margin are made to evaluate the margins. This method is suited for large poorly defined superficial tumors in the genital, perineal, and perianal regions, where a frozen section study would be slow and burdensome for the patient and surgeon. Keeping the wound open for several days also risks infection. Generally, the treatment of these lesions is complex, and the literature postulates MMS as the best therapeutic option. Many times, these tumors can be cured initially, while recurrences that occur over time are more challenging to eradicate.

The inclusion criteria are extensive epithelial tumors of the genital region with ill-defined clinical borders, macular, without nodular areas suggesting a phase of vertical growth, at least in the peripheral radial component. Deferred Lateral margin control is indicated in EMPD and vulvar melanoma, which often has a poorly defined lentiginous growth pattern. Specifically, immunohistochemical stains are required to clearly delineate the margins. The ST is not considered a technique of choice for well-defined small tumors or papular or nodular tumors where, in addition to the superficial component, a deep component may exist.

**Figure 12:**
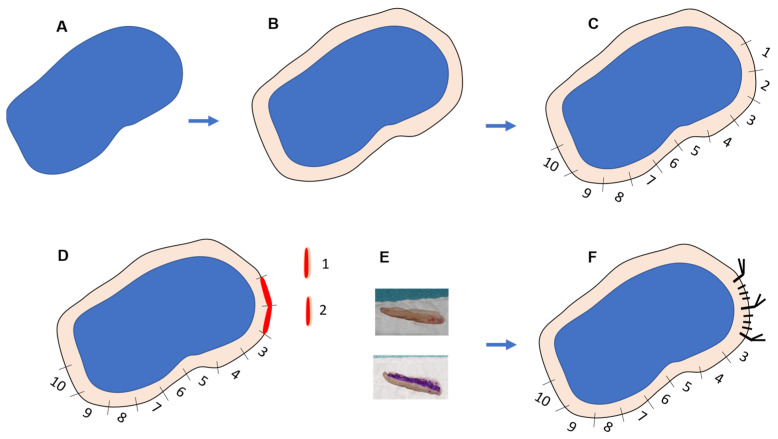
The tumor area is marked in blue using dermatoscopy and, if necessary, Wood’s light (**A**). A dermographic pencil outline is drawn around the tumor, extending a few millimeters beyond the clinical edge (**B**). The area is then divided into fragments measuring 1.5 to 2 cm and numbered for easier tracking (**C**). A scalpel is used to make incisions along the notches separating the fragments. Each piece is then cut into strips approximately 2 mm wide and 20 mm long (**D**). The strips are immersed in formalin for fixation, following the standard protocol. The image shows one marked piece and another unmarked (**E**). The defect left by the excised pieces is sutured directly. At the junctions between fragments, a silk stitch with longer ends can be left to facilitate identification later. Additional suture marks may also be placed between the pieces (**F**).

Surgical excision requires meticulous collaboration with pathology due to the high number of pieces and their orientation. It is crucial to photograph or draw the numbered pieces so the pathologist can accurately report and correlate them with the marks left on the patient (Figure 12). The fragments are sent to the pathology laboratory. They are usually pieces about 2 mm wide and no more than 20 mm long. The pathologist must place the piece in the cassette to facilitate a cut that studies the epidermis and dermis along the entire length of the fragment. The technique is easy to reproduce. Orientation of the pieces is not necessary, as there is only a real possibility of studying 2 edges, the outer face closer to healthy skin and the inner face, closer to the tumor. If the tumor is found in any of the cuts, it is considered that the margin is affected, regardless of whether the outer or inner margin has been analyzed. If the orientation of the pieces is desired, the outer face could be stained and indicated to the pathologist as the one to be analyzed.

Ideally, histological study should be performed promptly to avoid losing or erasing patient marks or tumor growth beyond them. Timing should be negotiated with the pathology lab, and postponing the second surgery for more than a week should be avoided. If positive margins are found, the procedure is repeated 5 mm beyond the involved area and sutured until a tumor-free perimeter is achieved. The need for a second paraffin pass depends on the location and extent of the margins; a few margins with minor involvement can be expanded or closed with Burow’s triangles (Figure 3B).

Conceptually, this is not conventional MMS or three-dimensional histology, as only lateral margins are assessed, not the depth. This approach is indicated for tumors that in their initial phase are only limited to the epidermis or the dermo-epidermal junction but do not invade the dermis or subcutaneous tissue, as seen in EMPD and the lentiginous component of vulvar melanoma. Table 1 shows the differences between conventional Mohs surgery and the spaghetti technique.

## 3. Discussion

Extramammary Paget’s disease and vulvar lentiginous melanoma are often extensive lesions, frequently ill-defined, located in the folds of the genital region, which for conventional Mohs surgery (frozen section) require many cuts and stains, meaning a lot of processing time, which ultimately becomes tedious for the pathologist, the Mohs surgeon, and the patient.

The literature indicates that approximately 2% of all female melanomas occur in the vulva. Surgical treatment remains the primary approach for localized vulvar melanomas. According to the National Comprehensive Cancer Network and the European Society for Medical Oncology guidelines for cutaneous melanoma, the recommended surgical margins depending on tumor thickness: 0.5–1 cm for melanoma in situ, 1 cm for invasive melanoma with Breslow thickness ≤1 mm, 1–2 cm for Breslow thickness between 1.01 and 2 mm, and 2 cm for Breslow thickness >2 mm. The same margins should be applied to vulvar melanoma. Immunotherapy with nivolumab or pembrolizumab should be offered to patients with resected stage IIB/C BRAF wild-type vulvar melanomas, while either of these agents, or a combination of dabrafenib and trametinib may be considered for BRAF-mutant cases. Notably, c-KIT mutations are present in more than 20% of vulvar melanomas, suggesting that tyrosine kinase inhibitors could be a viable option for recurrent disease. Several ongoing clinical trials may provide further direction for adjuvant and neoadjuvant treatment of mucosal melanomas in the near future (www.clinicaltrials.gov).

Only one prospective study on vulvar melanoma was conducted to date [16], along with retrospective data, suggesting that more radical vulvar surgeries, such as primary vulvectomy, do not yield better oncologic outcomes compared to local excision with the aforementioned margins [3]. Instead, they are associated with a higher rate of complications, including urinary incontinence and sexual dysfunction [17]. The MMS technique is appealing as it provides a stepwise method for confirming clear margins, potentially allowing for the avoidance of radical excision or vulvectomy. However, the current literature only reports the use of MMS as a treatment for vulvar melanoma in situ [5] in a single patient and in three male patients with penile melanoma [18,19]. Additional studies are necessary to determine whether ST surgery offers further benefits, such as reduced morbidity, faster recovery, and preservation of urinary and sexual functions. Vulvar melanoma in situ often occurs in postmenopausal women who may not regularly visit gynecologists. As a result, dermatologists can serve as the first line of defense in detection and play a crucial role in both the diagnosis and treatment of this condition.

Deferred MMS (in paraffin) has been proposed for excising tumors with complex interpretations in frozen sections, such as dermatofibrosarcoma protuberans [20,21], the lentiginous component of melanoma [22,23], extensive tumors like EMPD [24], and infiltrative tumors like squamous cell carcinoma [25]. These techniques, described in the literature as deferred Mohs surgery, slow Mohs [26], 3D histological study [27], complete circumferential peripheral and deep margin assessment with permanent sections, muffin technique [28], ‘‘square’’ procedure [29], Tübingen sections [30], rapid paraffin sections, or marginal and central staged excision using paraffin sections takes days or weeks, during which time open wounds in the genital region are at risk of infection and can have significant functional and psychological impacts. Other options are paraffin sections with tissue mapping before delayed surgical closure. Among the latter include the Perimeter technique [31] and the ST [15]; both involve excising lateral margins first and analyzing the perimeter fragments while preserving the tumor center for excision once the lateral margins are clear. The ST, similar to the Perimeter but with circumferential lateral margins, aims to define the shape and extent of the tumor, determining the resection shape and size for a second-stage reconstruction.

At least six studies [32,33,34,35,36,37] have analyzed the outcomes following the surgical excision of more than 100 LM or LMM using the Perimeter technique, demonstrating a lower recurrence rate and improved survival compared to histological assessment and conventional surgery. In the largest series published using this technique, a single-center retrospective study of 293 patients (225 LMs and 68 LMMs), the recurrence rate was 1.7% with a mean follow-up of 32.3 months [36]. In a prospective single-center study involving 292 LMMs (136 treated with paraffin MMS—the Perimeter technique—and 156 with conventional histology), the highest recurrence and melanoma-related mortality rates were observed in the conventional histology group [34]. With the ST described later, similar results have been obtained. In the original study, 21 patients were examined (16 LMs and 5 acral lentiginous melanomas); after a median follow-up of 25.36 months, the local control rate was 95.24%, with one case (4.76%) of invasive recurrence in transit. In another retrospective single-center study, involving 16 melanoma in situ and 15 invasive melanoma cases, only one patient (3%) experienced recurrence after a mean follow-up of 31 months [38]. A recent study shows that both wide local excision (WLE) and ST are appropriate surgical approaches for LM on the head and neck region [39].

Standard 5 mm margins for lentiginous melanoma are often insufficient for clear margins, and larger excision margins (10 mm) can lead to significant morbidity. The anatomical location, patient’s age, and comorbidities must also be considered. The clinical limits of the lentiginous component are often misleading and underestimated, as shown by a mean of 1.55 (up to 4) successive samplings of ‘‘spaghetti’’ before a tumor-free strip is found (15). This is in line with previous studies in LM showing an average of 1.67 (up to 5) stages in staged surgery of LM [40].

The ST offers several advantages, including comprehensive longitudinal dermatopathological control of the periphery. Tumors with poorly defined transition areas between healthy skin and tumor cells, corresponding by extrapolation to the field cancerization of squamous cell carcinoma/actinic keratoses, often require specific immunohistochemistry studies (S-100, Sox-100, PRAME) in melanoma [41] and cytokeratin 7 in EMPD [42].

Extramammary Paget’s disease of the genital region can exhibit different behaviors and evolution in men and women due to their specific anatomical characteristics. In women, the involvement often crosses the midline (central vulvar disease), is symmetrical, and is much more difficult to treat surgically than in men. Mohs surgery with curative intent is more realistic for genital involvement in men (scrotal involvement without perianal extension), while in women, when the involvement is closer to the midline (more periorificial), the chances of surgical cure decreases and morbidity increases. For this reason, more conservative treatments are sometimes considered in women than in men, such as imiquimod and photodynamic therapy. Another conservative option is radiotherapy, which is not without side effects and has a complexity of application due to anatomical localization.

A recent systematic review analyzes 209 articles, comprising 4133 patients [43]. The mean age was 70.3 years (range: 30–92) with 79.5% (2096/2637) males and 20.5% (541/2637) females. The most common regions included the pubic region (46.1%, 2004/4346) and scrotum (13.1%, 569/4346). There were 4069 (98.5%) and 64 (1.5%) cases of primary and secondary EMPD, respectively. For primary EMPD (*n* = 4390 treatments), the most common therapies included surgery [73.6%, 3233/4390; excision (*n* = 2795), MMS (*n* = 438)] and topical chemotherapy [11.3%, 496/4390; imiquimod (*n* = 461), 5-fluorouracil (*n* = 23), and ingenol mebutate (*n* = 12)]. Complete resolution (CR) was observed most frequently with treatment regimens including excision (79.2%, 2215/2795), MMS (95.2%, 417/438), and imiquimod (59.2%, 273/461). Recurrence was noted with 564 (12.8%) treatments, commonly involving excision (59.7%, 336/563) and imiquimod (11.4%, 64/563).

Nowadays, it is considered that EMPD, which spreads intraepidermally with poorly defined foci, shows lower recurrence rates with MMS. A 2017 cohort study found MMS recurrence rates of 11% compared to 36% with WLE, though this finding was not statistically significant. A recent meta-analysis reported that patients had a 2.67 times higher chance of local recurrence after WLE than MMS (95% CI: 1.47, 4.85; *p* = 0.0001). There are only four references in the literature regarding the treatment of EMPD using paraffin MMS. The largest study (*n* = 25) reported a 28% recurrence rate over an average follow-up of 4 years, using the Tübingen technique [44]. Hendi et al. [13], in a retrospective single-center study employing the Perimeter technique, reported a recurrence rate of 16% for primary disease and 50% for recurrent disease. The five-year tumor-free survival rates were 80% for primary tumors and 56% for recurrent tumors. Thomas et al. [45], in another retrospective single-center study, though without specifying the technique used, reported no recurrences during a 34-month follow-up period. Finally, Rodríguez Jiménez et al. [46], in a multicenter prospective cohort study conducted in Spain using various paraffin MMS techniques, observed no recurrences during a median follow-up of 0.9 years.

The ST can be combined with other margin delimitation tests, such as photodynamic diagnosis, Wood’s light examination, dermoscopy for defining the lentiginous component of melanoma (Collerette technique) [38], or confocal microscopy for LMM [47,48,49] and EMPD [50].

Dermatoscopy allows for a better evaluation than the naked eye of many epithelial neoplasms, especially pigmented lesions, clearly defining the clinical borders of the lesion. Wood’s light can assist the dermatologist in determining the clinical borders of an LMM or lentiginous melanoma. The natural tendency of melanin to absorb radiation in the UV range is utilized by Wood’s light examination; cutaneous lesions with a higher concentration of epidermal melanin will appear darker in contrast to the surrounding normal skin, while lesions with decreased melanin will appear brighter [51]. A recent article describes how dermoscopy with near-ultraviolet light highlights the demarcation of melanin distribution in cutaneous melanoma [52].

**Figure 13:**
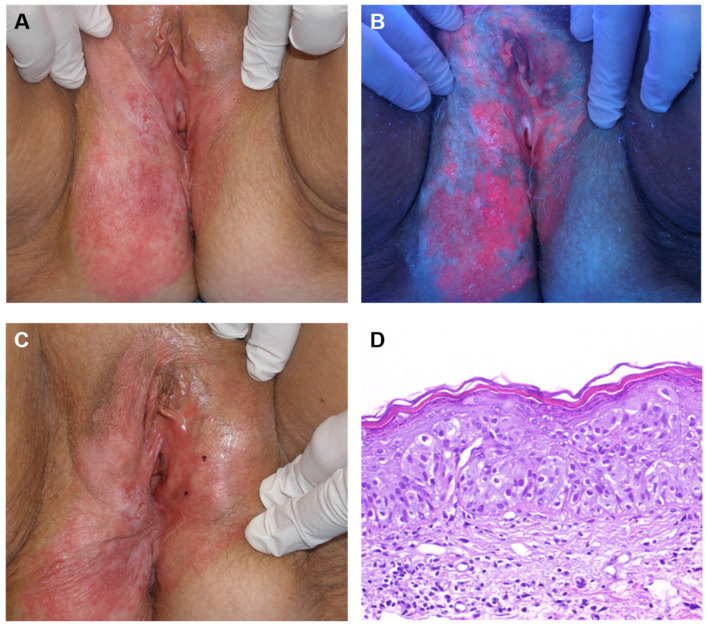
A 76-year-old woman with extramammary Paget’s disease primarily affecting the right vulva, with uncertain involvement of the left vulva (**A**). Photodynamic diagnosis: incubation with methylaminolevulinate cream for 2 h followed by illumination with Wood’s light. Coral red fluorescence is observed in both the right and left vulva (**B**). A black dot is marked to biopsy a clinically suspicious area with a positive photodynamic diagnosis (**C**). Histological confirmation of Paget’s disease in the left vulva (hematoxylin and eosin) (**D**).

Photodynamic diagnosis and reflectance confocal microscopy examination have been shown to be efficacious in tumor diagnoses (Figure 13). When it comes to determining the invasive margins of EMPD, photodynamic diagnosis and photodynamic diagnosis plus reflectance confocal microscopy (RCM) were found to be superior to observations made with the naked eye, while photodynamic diagnosis plus RCM was superior to photodynamic diagnosis alone [53]. A more recent approach is the use of in vivo presurgical mapping by RCM, which can identify subclinical LM beyond the recommended surgical margins [54].

Among the limitations of ST are the difficulty in ensuring complete excision without vertical margin assessment, and the need for precise paraffin histology. The essential difference between frozen sections and formalin-fixed sections is the greater use of an automated laboratory system to process the smaller size and greater number of blocks produced using the latter process. A formalin-fixed tissue Mohs service requires less technician time than a frozen section service. Theoretical limitations of the ST include difficulties in interpreting lateral sections and estimating differences between tumoral and normal melanocytes in sun-damaged skin. However, paraffin sections with immunohistochemistry are more reliable than frozen sections. The risk of tumor seeding is minimal, as the technique is used on the in situ part of lentiginous melanoma, with surgery performed after the last positive strip and a few weeks later.

Another limitation of the study is the sample size. Large multicentric studies are needed to evaluate the effectiveness of ST in extensive epithelial tumors of the genital region.

In summary, the ST preserves healthy tissue and is particularly useful for extensive or poorly defined cutaneous tumors in the genital region. Unlike traditional MMS techniques, the ST can be performed by any surgeon and pathologist without additional training and is more comfortable for patients, avoiding prolonged open wounds during multiple steps of tumor excision. The implementation of new non-invasive techniques for margin delineation, or the improvement in those already known, can help reduce the number of surgical passes to make treatments faster and more efficient with lower morbidity.

## Figures and Tables

**Table 1:** Mohs surgery vs. the Spaguetti technique. * The waiting time between the different stages of Mohs surgery is included.

	Mohs Surgery	Spaguetti Technique
Patient satisfaction	Good	Good
Number of days the patient enters the operating room	Almost always one day	Variable
Total duration of treatment	Longer (one time only) *	Shorter (sum of several times)
Quality of the analyzed tissue	Good	Best
Possibility of artifacts in sample processing (false negatives)	Low	Very low
Possibility of studying larges pieces	Limited	Adequate
Immunohistochemistry techniques	Limited	Widely available
Possibility of planning a surgical closure	At the time	In a planned way
Need for specialized dermatological and patological equipment	Yes	No

## Data Availability

The original contributions presented in this study are included in the article. Further inquiries can be directed to the corresponding author.
